# Formulation and Characterization of Quercetin-loaded Oil in Water Nanoemulsion and Evaluation of Hypocholesterolemic Activity in Rats

**DOI:** 10.3390/nu11020244

**Published:** 2019-01-22

**Authors:** Hye-Yeon Son, Mak-Soon Lee, Eugene Chang, Seog-Young Kim, Bori Kang, Hyunmi Ko, In-Hwan Kim, Qixin Zhong, Young-Hee Jo, Chong-Tai Kim, Yangha Kim

**Affiliations:** 1 Department of Nutritional Science and Food Management, Ewha Womans University, Seoul 03760, Korea; shyfree@gmail.com (H.-Y.S.); troph@hanmail.net (M.-S.L.); eugenics77@hotmail.com (E.C.); saraha9390@gmail.com (S.-Y.K.); pasodi00@naver.com (B.K.); commi801@naver.com (H.K.); 2 Department of Integrated Biomedical and Life Sciences, Korea University, Seoul 02841, Korea; k610in@korea.ac.kr; 3 Department of Food Science, University of Tennessee, Knoxville, TN 37996-6196, USA; qzhong@utk.edu; 4 Kolmar BNH CO., LTd 2-15, Sandan-gil, Jeonui-myeon, Sejong-si 30003, Korea; cho6452@naver.com; 5 R&D Center, EastHill Corporation, Gwonseon-gu, Suwon-si, Gyeonggi-do 16642, Korea

**Keywords:** quercetin, nanoemulsion, pH, stability, hypocholesterolemic activity

## Abstract

Due to poor water solubility and high susceptibility to chemical degradation, the applications of quercetin have been limited. This study investigated the effects of pH on the formation of quercetin-loaded nanoemulsion (NQ) and compared the hypocholesterolemic activity between quercetin and NQ to utilize the quercetin as functional food ingredient. NQ particle size exhibited a range of 207–289 nm with polydispersity index range (<0.47). The encapsulation efficiency increased stepwise from 56 to 92% as the pH increased from 4.0 to 9.0. Good stability of NQ was achieved in the pH range of 6.5–9.0 during 3-month storage at 21 and 37 °C. NQ displayed higher efficacy in reducing serum and hepatic cholesterol levels and increasing the release of bile acid into feces in rats fed high-cholesterol diet, compared to quercetin alone. NQ upregulated hepatic gene expression involved in bile acid synthesis and cholesterol efflux, such as cholesterol 7 alpha-hydroxylase (CYP7A1), liver X receptor alpha (LXRα), ATP-binding cassette transporter A1 (ABCA1) and ATP-binding cassette sub-family G member 1 (ABCG1). These results suggest at least partial involvement of hepatic bile acid synthesis and fecal cholesterol excretion in nanoemulsion quercetin-mediated beneficial effect on lipid abnormalities.

## 1. Introduction

Cardiovascular disease (CVD) remains the leading cause of death in the world [1]. A major risk factor for CVD is dyslipidemia, characterized by elevated plasma total cholesterol (TC) and their carrying lipoproteins and decreased HDL-cholesterol (HDL-C) [2]. Given this close link between dyslipidemia and CVD, a strategy to decrease CVD is to treat or improve blood cholesterol levels [3]. Natural food materials such as cocoa [4], curcumin [5,6,7], garlic [8], ginsenoside [9], hesperetin [10] and green tea [11,12] have been shown lipid-lowering effects by modulating hepatic cholesterol metabolism or intestinal fat absorption. Therefore, it is of great interest to their potentials as a future novel natural resource and a functional ingredient in food and pharmaceutical formations.

Plant polyphenols or flavonoid, rich in vegetables and fruits has been suggested to be the candidates for decreasing cardiovascular disease risk [13,14]. One of the most abundant flavonoids, quercetin (3,3′,4′,5,7-pentahydroxyflavone) is present in fruits and vegetables such as blood orange juice, red berry skin, prickly pear, red onions, apples, berries, citrus fruits, tea and red wine [15,16,17,18]. Numerous studies have demonstrated the anti-oxidant, anti-inflammatory, anti-hypertensive and anti-platelet properties of quercetin [19,20,21,22]. Still, lipid lowering effect of quercetin has not been fully elucidated. Therefore, the use of quercetin as nutraceutical ingredient has been interested. However, the applications of quercetin have been limited due to its poor water solubility and high susceptibility to chemical degradation upon exposure to alkaline and heat condition. These limitations lead to very low oral bioavailability and gastrointestinal absorption [23,24].

Several emulsification methods have been introduced to improve dispersibility and bioavailability through delivery systems. Quercetin-loaded microemulsions consisting of quercetin, oil, surfactant and emulsifier leads to significant increases of solubility and absorption parameters in rat intestine [23]. Still, alkaline conditions and heat exposure may cause rapid degradation of quercetin in microemulsion, thus affecting product processing and shelf life [25,26]. For the delivery of lipophilic bioactive compounds, oil-in-water (O/W) emulsion can be generated in a way of self-assembly emulsification using isotropic mixing oil, surfactant and co-surfactant in aqueous condition under agitation [27]. Simple oil-in-water emulsion and nanoemulsion can become thermodynamically unstable and finally degraded, because they consist of two immiscible liquids and emulsification increases interfacial area [28]. Therefore, it is critical to select appropriate emulsifies for the successful formation of emulsion, which can rapidly absorb onto the surface of oil droplets and effectively reduce the interfacial tension between oil and water phases. Food biopolymers such as lecithin, sodium caseinate and decaglycerol monooleate could be employed to generate stable emulsion-based delivery system [29]. Furthermore, food-grade biopolymers such as polysaccharides (alginate, chitosan, pectin, gum arabic) and proteins (casein, gelatin, whey) could be useful to generate novel nanoparticle delivery systems by promoting self-association or aggregation of single biopolymers or mixed biopolymer systems [30]. Compared to single-layered nanoemulsion, double-layer nanoemulsion improves the stability and physicochemical properties in our previous study [31]. In addition, pH is one of the most important factors considering structural stability during food processing, storage, transportation and utilization. However, there is no study reporting characterization and stabilization of quercetin-entrapped double-layered nanoemulsion over a wide range of pH during long storage duration. 

In the present study, we newly developed quercetin-loaded double layer nanoemulsion delivery systems using complexation and self-assembly with caprylic/capric triglyceride (Captex^®^ 355, Abitec Co., Janeville, WI, USA), polyoxyethylene sorbitan monooleate (Tween 80, Sigma-Aldrich Chemical Company, St. Louis, MI, USA), sodium alginate and soy lecithin in water. The first purpose of the present study was to characterize physicochemical properties of double layer oil-in-water nanoemulsion encapsulated with quercetin over a wide range of pH or different storage periods. The second objective of this study was to investigate the hypocholesterolemic effects of a nanosized quercetin emulsion in rats fed a high-cholesterol diet.

## 2. Materials and Methods 

### 2.1. Materials 

Quercetin (purity >95%) was purchased from Acros Organics Company (Fisher Scientific, Pittsburgh, PA, USA). Caprylic/capric triglyceride (Captex^®^ 355) was purchased from Abitec Co. (Janeville, WI, USA). Soy lecithin was purchased from IFC Solutions (Linden, NJ, USA). Polyoxyethylene sorbitan monooleate (Tween^®^ 80) and sodium alginate from algae were purchased from Sigma-Aldrich Chemical Company (St. Louis, MI, USA). All chemical reagents used in this study were of the highest purity commercially available.

### 2.2. Formation of Quercetin Nanoemulsions

To prepare the nanoemulsion, a primary emulsion was formulated by mixing 300 mg of quercetin and 3 g of Captex^®^ 355. The mixture was dispersed with 3 g of Tween^®^ 80 using stirrer for 1 h at 65 °C. The mixture was homogenized with a high-speed homogenizer (Ultra-Turrax T-25, Janhke & Kunkel Gmbh and Co KG, Staufen, Germany) at 12,000 rpm for 4 min. After homogenization, 6 g of primary emulsion was added to a previously prepared mixture of 15 g of 5% sodium alginate and 1.5 g of soy lecithin (IFC Solutions Inc., Linden, NJ, USA) and mixed on a Vortex mixer at 600 rpm for 3 min. For nanoemulsion preparation, 0.5 g of the above primary emulsion mixture was dispersed into 100 mL of pure water and the pH was adjusted to range from 4.5 to 9.0 with 0.1 N HCl and 0.1 N NaOH under mild stirring for 3 h. Subsequently, the nanoemulsions were stabilized for 24 h at room temperature.

For the formation of animal diets. 120 g primary emulsion was formulated and dispersed into 8 kg of pure water and adjusted the pH to 8 and under a mild stirring for 3 h for the preparation of quercetin-loaded nanoemulsion, as described above. After 24 h stabilization at room temperature, quercetin nanoemulsion was freeze dried before use. 

### 2.3. Characterization of Nanoemulsion

#### 2.3.1. Determination of Particle Size and Polydispersity Index (PDI)

The particle size and PDI of each quercetin nanoemulsion were determined by photon correlation spectroscopy with a Nanotrac 250 (Microtrac, Inc., Montgomeryville, PA, USA). Light scattering was monitored at 25 °C and an angle of 90 °C. Each sample was measured in triplicate.

#### 2.3.2. Determination of the zeta (ζ) Potential

The ζ potential of each quercetin nanoemulsion was determined by placing 1 mL sample in a disposable ζ cuvette (1 × 1 × 4 cm). The cuvette was inserted into the measurement chamber of the particle electrophoresis instrument (Zetasizer Nanoseries ZS, Malvern Instrument, Worcestershire, UK) and equilibrated to 25 °C in the Peltier-controlled cuvette holder 2 min before use. The zeta (ζ) potential was determined by measuring the direction and velocity that the nanoemulsion moved in an applied electric field at 633 nm for 1 min. The measurement of each sample was repeated in triplicate. 

#### 2.3.3. Determination of Encapsulation Efficiency

Each nanoemulsion was centrifuged at 4000× *g* for 10 min to separate unloaded quercetin and the supernatant was again centrifuged twice, followed by filtration through a 0.45-μm membrane filter. After the precipitate was dissolved in methanol, the sample was prefiltered with a 0.45-μm Millipore filter disk and degassed. For measurement of quercetin concentration, HPLC methods were employed on an Agilent 1260 Infinity Quaternary LC system (Agilent Technologies Inc., Santa Clara, CA, USA) equipped with an Agilent 1260 autosampler and an Agilent 1260 UV. Each sample solution was injected into an Agilent Eclipse XDB-C18 column (250 mm × 4.6 μm × 5 μm) (Zorbax, Agilent Technologies Inc., Santa Clara, CA, USA). The mobile phase system consisted of methanol and double-distilled water (70:30, *v*/*v*) acidified with 0.1% formic acid. The flow rate was 0.8 mL/min and the wavelength was set at 368 nm. The percentage of quercetin within the prepared nanoemulsion was calculated. The encapsulation efficiency was then calculated by Equation (1).

(1) $$\mathrm{Encapsulation}\;\mathrm{efficacy}\;\mathrm{of}\;\mathrm{the}\;\mathrm{prepared}\;\mathrm{quercetin}\;\mathrm{nanoemulsion}\;\left(\%\right)\;=\frac{\mathrm{Total}\;\mathrm{quercetin}\;\mathrm{added}\;-\;\mathrm{Querecetin}\;\mathrm{in}\;\mathrm{precipitate}}{\mathrm{Total}\;\mathrm{quercetin}\;\mathrm{added}}\;\times \;100$$

#### 2.3.4. Measurement of Morphology

The morphology of each nanoemulsion was examined with a transmission electron microscope (TEM) (Tecnai G2 F20, FEI Company, Eindhoven, The Netherlands). For negative staining, 7 μL of the nanoemulsion was placed on a carbon-coated copper grid and washed once with ultrapure water. Then, the sample was stained immediately with 7 μL of 2% uranyl acetate in ultrapure water, washed with water again and then air-dried. Images were acquired at 200 kV and the scales were calibrated with a grating replica 3-mm grid. A BioScan camera (Model 792, Gatan, Inc, Pleasanton, CA, USA) and Digital-MicrographTM software (version 3.4; Gatan, Inc, Pleasanton, CA, USA) were used to capture images.

#### 2.3.5. Long-term Physical Stability Studies

The nanoemulsions were kept in siliconized glass vials with addition of sodium azide (0.02% (*w*/*w*)) as antibacterial agent and were stored at 21 ± 2 °C and 37± 2 °C for 3 months. The particle size, PDI and zeta potential of nanoemulsions were measured under different storage intervals for a period of 3 months. The measurement of each sample was repeated in triplicate.

### 2.4. Animals and Experimental Design

Six-week old male Sprague Dawley rats, weighing 180–200 g were purchased (Doo Yeol Biotech, Seoul, Korea). Each rat was individually housed in a cage with controlled environment; temperature (22 ± 2 °C), humidity (55 ± 5%) and lighting (12 h light/dark cycle). All experiment methods and care were approved by Institutional Animal Care and Use Committee (IACUC) of Ewha Womans University (IACUC No. 16-015). After 1 week of acclimatization with free access to water and normal chow diet, the animals were randomly divided into 6 groups (*n* = 8/group) and fed a normal chow diet (NOR), high cholesterol diet containing 1% cholesterol and 0.5% cholic acid (HC), HC containing 0.05% quercetin (LQ) or 0.1% quercetin (HQ) and 0.05% quercetin nanoemulsion (LNQ) or 0.1% quercetin nanoemulsion (HNQ) (Table S1). Feces were collected during the last three days of the experiment and stored at −40 °C. At the end of 4 weeks, rats were fasted overnight and were anesthetized with mixtures of Zoletil 50 (Virbac Laboratories, Carros, France) and Rompun (Bayer Korea, Seoul, Korea). Blood was collected by cardiac puncture, centrifuged at 1516× *g* for 20 min at 4 °C to obtain serum and stored at −70 °C. Liver and white adipose tissue (epididymal; WAT) were excised and stored at −70 °C until further analysis.

### 2.5. Evaluation of Nanoemulsion Hypocholesterolemic Activity

#### 2.5.1. Serum Biochemical Measurements

Serum concentrations of triglycerides (TG), total cholesterol (TC), high-density lipoprotein cholesterol (HDL-C), glutamic oxaloacetic transaminase (GOT) and glutamic pyruvic transaminase (GPT) were determined by enzymatic colorimetric methods with commercial kits (Asan Pharmaceutical, Seoul, Korea) in accordance with the manufacturer’s instructions. The low-density lipoprotein cholesterol (LDL-C) concentration was calculated by the Friedewald formula [32]. 

LDL-C = TC − HDL-C − (TG/5)(2)

#### 2.5.2. Hepatic and Fecal Lipid Analysis

Hepatic and fecal lipids were extracted by the method of Bligh and Dyer with a slight modification, as previously described [33]. Briefly, liver and feces were homogenized in 1.5 mL 0.9% saline and 7.5 mL of methanol:chloroform (2:1, *v*:*v*) was added to the homogenates. The mixture was shaken for 10 min and centrifuged (2000× *g*, 20 min). The lower chloroform phase was collected and the lipid in this phase was dried and weighed. TG and TC levels in the liver and feces were measured by enzymatic colorimetric methods with commercial kits as described above. 

#### 2.5.3. Bile Acid Analysis

Fecal lipid extracts were diluted in mixture of n-Hexane and isopropanol (3:2, *v*/*v*) before total bile acids (TBA) measurement. The total bile acids levels in fecal lipid sample were determined with a total bile acid assay kit (Wako Pure Chemical Industries Ltd., Osaka, Japan) in accordance with the manufacturer’s instruction. Reacted substrates create end color products considered to be directly proportional to concentration of TBA.

#### 2.5.4. Immunohistochemistry (IHC) Staining

Liver tissues from the rats were fixed in 10% formalin and embedded in paraffin. After deparaffinization and rehydration of the tissues, antigen retrieval was performed with 0.01 M sodium citrate buffer (pH 6.0) in a water bath to 95°C for 40 min. The tissue sections were incubated in peroxide blocking buffer (ScyTek, Logan, UT, USA) for 10 min. Sections were then incubated with goat monoclonal CYP7A1 antibody (C-20, Santa Cruz Biotechnology, Santa Cruz, CA, USA) and analyzed with a Polink-2 Plus HRP Anti-rat DAB Detection kit (Golden Bridge International, Inc., Bothell, WA, USA) according to the manufacturer’s instructions. The sections were stained with diaminobenzidine (Golden Bridge International, Inc., Bothell, WA, USA) and counterstained with Mayer hematoxylin (ScyTek, Logan, UT, USA). The images were visualized by microscopy (Olympus, Tokyo, Japan).

#### 2.5.5. Measurement of Hepatic Microsomal CYP7A1 Activity

Hepatic microsomal CYP7A1 protein activity was determined with a rat CYP7A1 ELISA kit (Biomatik, Wilmington, DE, USA) according to the manufacturer’s protocol. 0.2 g frozen liver samples were weighed and homogenized in homogenization buffer by 10–15 strokes in a tight-fitting Dounce homogenizer. Liver microsomes were extracted with a microsome isolation kit (Abcam, Cambridge, MA, USA). The microsomal protein content was determined with a BCA protein assay kit (Thermo Scientific, Waltham, MA, USA). The CYP7A1 activity of each sample was normalized to the respective microsomal protein concentration and expressed as ng/g microsomal protein.

#### 2.5.6. Real-time Quantitative Reverse-transcription Polymerase Chain Reaction (qRT-PCR)

Total RNA was isolated with TRIzol reagent (Invitrogen, Carlsbad, CA, USA). The cDNA was synthesized from 4 μg of RNA with M-MLV reverse transcriptase (Bioneer Co., Daejeon, Korea). qRT-PCR was performed with AccuPower 2X Greenstar qPCR MasterMix (-ROX Dye) (Bioneer Co., Daejeon, Korea) on a fluorometric thermal cycler (Corbett Research, Mortlake, NSW, Australia). Primers used in the present study are described in Table S2. Gene expression was calculated according to the comparative 2 ^−ΔΔCT^ method [34]. The ΔΔCt value for each sample was determined by calculating the difference between the Ct value of the target gene and the Ct value of the reference gene (β-actin). Values were expressed as fold change of the HC group.

### 2.6. Statistical Analysis 

All data are expressed as the mean ± standard error of the mean (SEM). Statistical analyses were conducted with SPSS software (version 21; IBM Corporation, Armonk, NY, USA). One-way analysis of variance (ANOVA) and Tukey’s multiple comparison tests were performed to determine the significance of differences. A *p*-value < 0.05 was set as the significance level.

## 3. Results

### 3.1. Effect of pH on Nanoemulsion Formation

A quercetin-loaded double layer oil-in-water nanoemulsion delivery system was developed by complexation and self-assembly with Captex^®^ 355. Tween 80, sodium alginate and soy lecithin over a broad pH range. Figure S1 illustrates the possible incorporation of quercetin in the oil-in-water nanoemulsion. Generally, food products and beverage exhibit broad range of pH, hence it is important to understand the influence of pH on the stability of nanoemulsion. Various nanoemulsions were prepared with different pH conditions as described above (Table 1, Figure S2A,B). Through the addition of acid, pH reductions to acidic pH in primary emulsions contribute to droplet aggregation [25]. Indeed, nanoemulsions prepared at pH between 4.0 and 6.0 were not transparent and exhibited undispersed, flocculated, clumped, separated and opaque patterns. In contrast, no flocculation was observed at pH between 6 and 7, suggesting that these emulsions were relatively stable to droplet aggregation. In addition, nanoemulsions formed at pH ranging from 6.5 to 9.0 exhibited dispersibility and transparency without flocculation, clumping, separation or opacity, demonstrating good stability (Table 1, Supplementary Figure S2). Quercetin-loaded nanoemulsions prepared at pH 6.5, 7.0, 7.5, 8.0, 8.5 and 9.0 were used in further experiments to study the morphology, encapsulation efficiency, particle size, zeta potential and long-term physical stability during 3-month storage.

### 3.2. Influence of pH on Nanoemulsion Physical Characteristics 

Table 1 shows the effects of pH on the particle size, PDI (an index to describe the degree of non-uniformity of a size distribution of particles) [35] and zeta potential of nanoemulsion. A monomodal particle size in the range of 207–289 nm was observed (Table 1). The particle sizes of the quercetin-loaded nanoemulsions at pH between 4.0 and 6.0 were relatively larger than those at pH between 6.5 and 9.0 (Table 1). At low pH, the nanoemulsions were highly unstable due to droplet aggregation growth and phase separation [29,36]. With regard to droplet growth, our results demonstrate that nanoquercetin prepared at pH above 6.0 could be relatively stable.

The quercetin-loaded oil-in-water nanoemulsions had acceptable PDIs less than 0.47 (Table 1). Similar to the particle sizes, the PDIs were lower at pH between 6.5 and 9.0 than at pH between 4.0 and 6.0. During the process of nanoemulsion incorporation, the liquid form must be converted to a dry powder form, termed as lyophilization. When the PDI remains the same before and after this lyophilization process, it shows the ability that the nanoemulsion distributes uniformly in the dispersion medium [37]. With regard to the unified droplet distribution, the relatively low PDI values at pH levels between 6.5 and 9.0 indicate that these pH values result in more stable nanodelivery systems by yielding monodisperse systems.

In the present study, three different regions of zeta potential were observed (Table 1): −22.1 to −45.8 mV (acidic pH, 4.0–5.5), −52.8 to −65.3 mV (neutral pH, 6.0–7.5) and −62.0 to −65.5 mV (alkaline pH, 8.0–9.0). In addition, at pHs of 4 and 5, the zeta potential was close to −30 mV, which is near the isoelectric point of sodium alginate. 

### 3.3. Encapsulation Efficiency and Morphology of Quercetin

**Figure 1:**
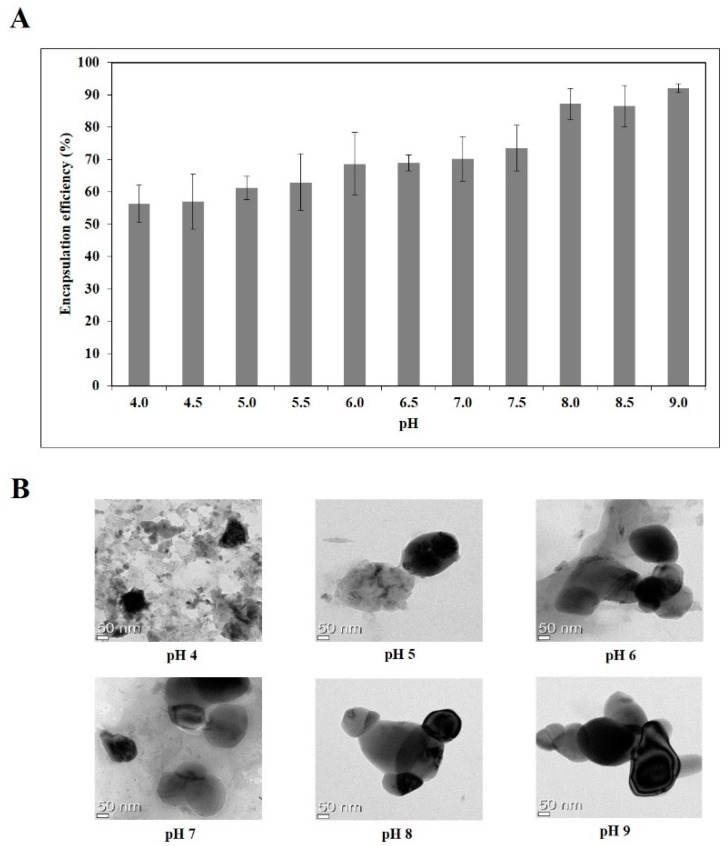
Encapsulation efficiency (**A**) and TEM images (**B**) of quercetin-loaded nanoemulsion with different pH.

Next, the amount of unloaded quercetin was determined after pretreatment of the nanoemulsions and the encapsulation efficiency of quercetin were evaluated. As shown in Figure 1A, encapsulation efficiency increased from 56 to 92% as the pH increased from 4.0 to 9.0. A positive correlation (*R*^2^ = 0.68) was found between zeta potential and encapsulation efficiency of the quercetin-loaded nanoemulsions (Figure S3), illustrating that these physical properties could be important factors for the formulation of quercetin-loaded nanoemulsions.

Double layer nanoemulsions prepared at pH of 4, 5, 6, 7, 8 and 9 were observed by TEM analysis (Figure 1B). Whereas the quercetin-loaded nanoemulsion at pH 4 tended to clump, probably due to the aggregation and gelation of sodium alginate and lecithin, the nanoemulsions at pH of 5, 6, 7, 8 and 9 had spherical or oval shapes and droplet-type nanoemulsion structures.

### 3.4. Long-term Storage Stability

**Figure 2:**
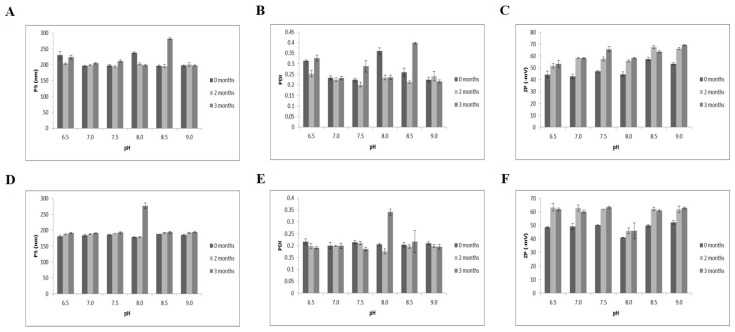
Physical stability of quercetin-loaded nanoemulsions for 3 months. (**A**–**C**) nanoemulsion storage at 21 °C; (**D**–**F**) storage at 37 °C. PDI, Polydispersity index; PS, particle size; ZP, zeta potential.

The long-term storage stabilities of the quercetin-loaded nanoemulsions were investigated at two different temperatures (21 and 37 °C) for 3 months based on particle size, PDI and zeta potential (Figure 2). At 21 and 37 °C for 3 months, the particle sizes of the nanoemulsions were 192–282 nm (Figure 2A) and 178–277 nm (Figure 2D), respectively. The PDIs ranged from 0.2–0.4 at both storage temperatures (Figure 2B,E). The range of zeta potentials increased from −42.8~−69.1 mV at 21 °C (Figure 2C) to −41~−63 mV at 37 °C (Figure 2F). Consistent with a study indicating that high temperature (>55 °C) and long-term storage lead to instability in casein-based nanoemulsions [29], storage of quercetin-loaded nanoemulsions with Captex^®^ 355, Tween 80, sodium alginate and soy lecithin for 3 months at 37 °C resulted in higher zeta potentials than storage at the same pH at 21 °C. However, no obvious changes in oil droplet growth or phase separation were observed according to pH level (Figure S2). Based on particle size, PDI, zeta potential, visual appearance, complexation and self-assembly, the nanoemulsions exhibited good physical stability at both 21 and 37 °C for 3 months over a broad pH range of 6.5–9.0.

### 3.5. Effects of Quercetin-loaded Quercetin on Safety in High Cholesterol-fed Rats

To investigate hypocholesterolemic effect of dietary quercetin-loaded nanoemulsion (NQ) in high-cholesterol diet-fed rats, nanosized quercetin emulsion was prepared at a pH of 8, selected from the study to investigate the effects of pH on the formation and characterization of NQ. In detail, used NQ in animal diets had 168.3 ± 15.6 nm mean particle size, −61.91 ± 5.64 mV zeta potential and 87.20 ± 4.73% loading capacity at pH 8. Figure S4 demonstrates the morphology of the quercetin nanoemulsion used in the animal study. Two dosages of either quercetin or nanoemulsion quercetin (0.05 or 0.1% *wt*/*wt*) were supplemented in high cholesterol diet and administered to rats for 4 weeks. 

First, we investigated the effects of dietary Q or NQ supplementation on liver toxicity and high-cholesterol diet-induced hepatomegaly. Liver tissue weight was about 1.5-fold higher in rats fed a high-cholesterol diet (HC) than in those fed an NOR diet, demonstrating that HC leads to liver hypertrophy. However, inclusion of either quercetin (LQ, HQ) or the quercetin-loaded nanoemulsion (LNQ, HNQ) in the HC diet did not change the liver weight from that on the HC diet (Table 2). To determine whether the usage of quercetin-loaded nanoemulsion is safe in rats, serum GOT and GPT activities were measured. There was no statistical different serum GOT and GPT activities among the groups (Table 2). 

### 3.6. Hypocholestrolemic Effect of Quercetin-Loaded Nanoemulsion 

#### 3.6.1. Body Weight, Energy Intake and Fat Accumulation

After 4 weeks of quercetin or nanoquercetin supplementation in high cholesterol diet, no significant differences in final body weight or body weight gain were observed. In addition, neither food intake nor food efficiency differed according to the experimental diets. The epididymal adipose tissue (WAT) weight also did not differ among the groups (Table 2). 

#### 3.6.2. Serum and Hepatic Lipid Profiles

**Figure 3:**
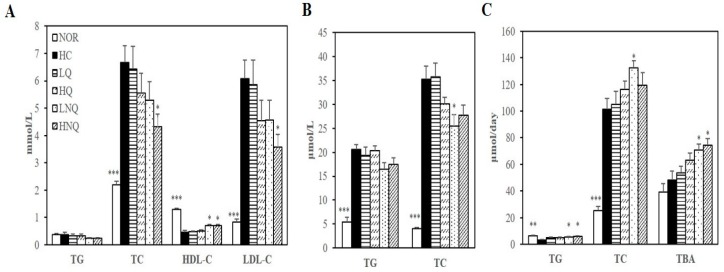
Effects of nanoemulsion quercetin on serum, hepatic and fecal lipid profiles. (**A**) Serum lipid profiles. LDL-C = TC − HDL-C − (TG/5). (**B**) Hepatic lipid profiles. (**C**) Fecal lipid profiles together with bile acid levels. Values are expressed as mean ± SEM (*n* = 8). * *p* < 0.05, ** *p* < 0.01, *** *p* < 0.001 versus HC group. HDL-C, HDL-cholesterol; LDL-C, LDL-cholesterol; TBA, total bile acid; TC, total cholesterol; TG, triglyceride. NOR: normal chow diet, HC: high cholesterol diet containing 1% cholesterol and 0.5% cholic acid, LQ: HC containing 0.05% quercetin, HQ: HC containing 0.1% quercetin, LNQ: HC containing 0.05% quercetin nanoemulsion, HNQ: HC containing 0.1% quercetin nanoemulsion.

Serum and hepatic lipid levels were not changed by quercetin (LQ, HQ; Figure 3A,B). In contrast to rats fed LQ and HQ diets, rats fed a high-cholesterol diet containing the quercetin-loaded nanoemulsion had lower serum TC and LDL-C levels and hepatic TC concentrations than rats fed the high-cholesterol diet. The HC-increased serum concentrations of TC and LDL-C were reduced by 35.3% and 41.2%, respectively, in the 0.1% quercetin nanoemulsion-supplemented group (HNQ) (*p* < 0.05) (Figure 3A). Moreover, the serum HDL-C concentrations of the LNQ- and HNQ-fed rats were 56.0% and 54.1% higher, respectively, than those of the HC-fed animals (Figure 3A). In addition, the quercetin nanoemulsion reduced hepatic TC level in a dose-dependent manner, reaching statistical significance at the 0.05% dose (LNQ) (*p* < 0.05) (Figure 3B). 

#### 3.6.3. Fecal Lipid Levels and Bile Acid Excretion

Fecal TG levels were significantly greater in rats fed either LNQ or HNQ than in rats fed the HC diet, increasing by 87.6% and 101.1%, respectively (*p* < 0.05) (Figure 3C). In addition, the quercetin nanoemulsion tended to increase fecal TC levels, reaching statistical significance at the 0.05% dose (LNQ) (*p* < 0.05) (Figure 3C). Moreover, fecal bile acids (measured by TBA levels) increased dose-dependently in the LNQ and HNQ groups compared with the HC group (*p* < 0.05) (Figure 3C). However, LQ or HQ did not change fecal lipid profiles and bile acid levels (Figure 3C). In this regard, it is possible that the quercetin nanoemulsion-reduced serum and hepatic lipid profiles of HC-diet fed rats were associated with increased fecal cholesterol excretion.

#### 3.6.4. Hepatic Gene Expression Related to Cholesterol Efflux

**Figure 4:**
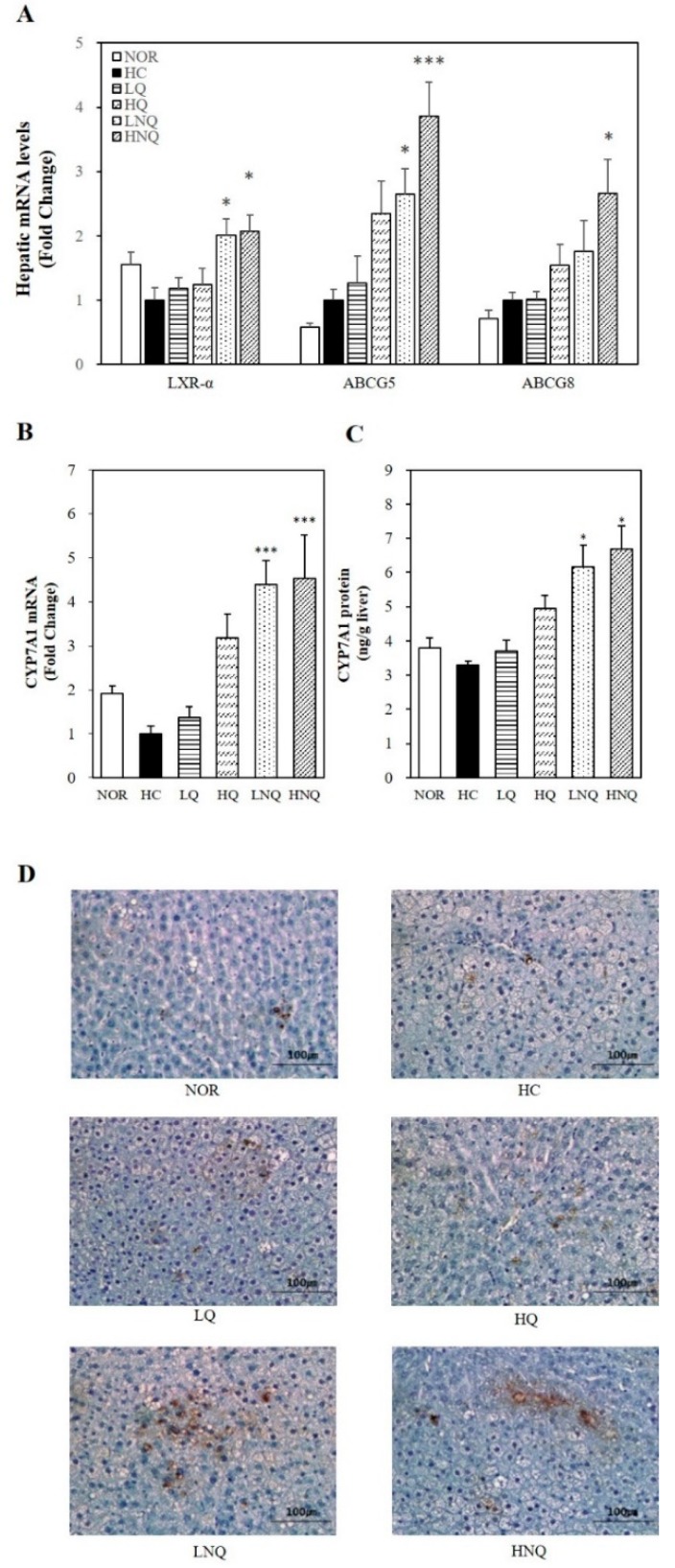
Influence of quercetin nanoemulsion on hepatic gene expression related to cholesterol efflux. mRNA levels of LXRα, ABCG5 and ABCG8 (**A**) and CYP7A1 (**B**) were measured by real-time qPCR and normalized to β-actin. The results were expressed as the fold change compared to HC. CYP7A1 enzyme activity was measured by a rat CYP7A1 ELISA kit and normalized to their relative microsomal protein contents (**C**). Immunohistochemistry staining for CYP7A1was performed using a diaminobenzidine-based staining technique (**D**). Values are expressed as means ± SEM (*n* = 8). * *p* < 0.05, *** *p* < 0.001 versus HC group. CYP7A1, Cholesterol 7α-hydroxylase. NOR: normal chow diet, HC: high cholesterol diet containing 1% cholesterol and 0.5% cholic acid, LQ: HC containing 0.05% quercetin, HQ: HC containing 0.1% quercetin, LNQ: HC containing 0.05% quercetin nanoemulsion, HNQ: HC containing 0.1% quercetin nanoemulsion.

In the current study, the mRNA levels of all the key regulators of cholesterol efflux in the form of fecal bile acids (LXRα, ABCG5 and ABCG8) were significantly upregulated by quercetin nanoemulsion supplementation on the high-cholesterol diet (*p* < 0.05) (Figure 4A). Thus, we suggest that the quercetin nanoemulsion may have improved the HC-induced hypercholesterolemic phenotype by promoting cholesterol efflux through the activation of LXRα and thus ABCG5/8 expression.

#### 3.6.5. Hepatic CYP7A1 Gene Expression and Microsomal Activity

To determine the effects of the quercetin nanoemulsion on bile acid synthesis, we measured the hepatic mRNA expression and activity of cholesterol 7 alpha-hydroxylase (CYP7A1), a rate-limiting enzyme of bile acid synthesis. Given the association between CYP7A1 and bile acid synthesis, the hepatic mRNA expression (Figure 4B), microsomal activity (Figure 4C) and protein levels (Figure 4D) of CYP7A1 were examined in the present study. The quercetin nanoemulsion significantly upregulated hepatic CYP7A1 mRNA expression and its microsomal activity. In addition, IHC revealed abundant CYP7A1 expression in the liver tissue of rats fed the quercetin nanoemulsion. These data suggest that the favorable effects of the quercetin nanoemulsion on HC diet-induced dyslipidemia may have been associated with increased hepatic CYP7A1 expression. 

## 4. Discussion

A large number of epidemiological studies have shown the close association between lipid abnormalities and an increased risk of CVD [2,38,39,40,41]. Conversion of cholesterol to bile acids in the liver and biliary cholesterol excretion to feces are the major pathway for cholesterol homeostasis by removing excess cholesterol from the body [42]. Beneficial effect of dietary quercetin on cholesterol metabolism has not been fully determined. In addition, usage of newly developed nanotechnology has been shown to improve health outcomes [43,44,45]. These led us to develop quercetin-loaded double layered oil-in-water nanoemulsion and investigate favorable effect of nanoemulsion quercetin on high cholesterol diet-induced hypercholesterolemia and fecal cholesterol excretion.

In the present study, the complexation and self-assembly with Captex^®^ 355, Tween 80, sodium alginate and soy lecithin has been employed to develop a quercetin-loaded double layer oil-in-water nanoemulsion techniques. Owing to a broad range of pH in food products and acidic pH-induced droplet growth and phase separation in primary emulsion formulation [25,29,36], the influence of pH on the stability of nanoemulsion was investigated. In regard to the appearance of nanoemulsion formation, nanoemulsions prepared at pH ranging from 4.0 to 6.0 were not transparent but undispersed, flocculated, clumped, separated and opaque. In contrast to acidic pH levels, pH between 6.5 and 9.0 did not induce the flocculation, clump, separation and opaque in emulsion formation. Next, the characterization of quercetin-loaded nanoemulsion were investigated by measuring particle size, PDI and zeta potential in different pH conditions. At a broad pH range (4.0–9.0), a monomodal nanoparticle size was between 207 and 289 nm and PDI were less than 0.47. The dissolution or dispersion to emulsion membrane layer makes quercetin partitioning into oil phase which leads to the changes of nanoemulsion droplet size. During the process of nanoemulsion incorporation, the conversion from liquid to dry powder, lyophilization occurs. When PDIs are same before and after this lyophilization, it indicates that nanoemulsion uniformly distributes in the dispersion medium [37]. Thus, smaller particle size and PDI indicates more stable nanodelivery ability. In this present study, pH between 4.0 and 6.0 had larger particle size and PDI of quercetin-loaded nanoemulsions, compared to pH ranging from 6.5 to 9.0. According to pH level, there were three different zeta potential sections; −22.1~−45.8 mV (pH 4.0–5.5), −52.8~−65.3 mV (pH 6.0–7.5) and −62.0~−65.5 mV (pH 8.0–9.0). For generating stable emulsion particles by electrostatic stabilization, zeta potential values ±30 mV as a minimum are preferable [46]. In the present study, zeta potential of nanoquercetin prepared at pH 4 or 5 was close to −30 mV, which were closed to isoelectric point of sodium alginate. Due to the attractive forces between particles resulting from electrosteric repulsion and van der Waals attraction, the reduced charge of sodium alginate-coated droplets at pH near the isoelectric point results in droplet aggregation [29]. Therefore, nanoquercetin prepared at pH 4 or 5 seems to be unstable. In addition, the encapsulation efficiency was increased from 56 to 92% according to pH increase from 4.0 to 9.0 showing positive correlation with zeta potential values. Furthermore, morphology of nanoemulsion was observed by TEM. Compared to a clump of nanoemulsion at pH 4, nanoemulsion prepared at pH above 5 and 21 °C had spherical or oval shapes and droplet-type structures. In addition, 3-month storage at 21 or 37 °C did not induce physical instability such as flocculation, clumping, separation and opacity over a broad pH range of 6.5–9.0. Based on the appearance and physicochemical properties of quercetin-loaded nanoemulsion and long term storage stability, nanoquercetin prepared at a wide range of pH (6.5–9.0) seems to be stable. 

Next, the hypocholesterolemic effects of the nanoquercetin was determined in in rats fed high-cholesterol diet. For the preparation of nanoquercetin in this animal study, a pH of 8 was selected from appearance, characterization and encapsulation efficiency of nanoemulsion formation. Newly formulated nanoquercetin used in animal study had 168.3 ± 15.6 nm particle size, −61.91 ± 5.64 mV zeta potential and 87.20 ± 4.73%. loading capacity at a pH of 8.0 were investigated. Compared to quercetin, 4-week supplementation of the nanoquercetin, NQ showed higher hypocholesterolemic efficiency in reducing HC diet-induced serum and hepatic lipid abnormalities and increasing the release of bile acid into the feces without changing body weight and food intake. The currently used dosages of quercetin and nanoemulsion quercetin (0.05% or 0.1% *wt*/*wt*) were determined as described in previous studies [43,44,45]. At a given dose, there was no difference in serum GOT and GPT concentrations and liver weight, illustrating two dosages of quercetin and nanoemulsion quercetin used in this study were well tolerated by the experimental animals. Cholesterol homeostasis is maintained by the balance between hepatic cholesterol biosynthesis and hepatic cholesterol catabolism [47]. In the maintenance of cholesterol homeostasis, bile acids are the end products of cholesterol catabolism and their biosynthesis and excretion to feces are involved in a reduction of excess hepatic cholesterol [48]. In the present study, quercetin (LQ, HQ) did not change serum, hepatic or fecal lipid levels, consistent with previous studies [49,50]. However, other animal studies have shown favorable effects of quercetin on lipid profiles [51,52,53]. A plausible explanation for these inconsistencies may be low hydrophilic characteristics of quercetin or different animal models. In contrast to quercetin, serum TC and LDL-C and hepatic TC concentrations in rats fed a high-cholesterol diet were significantly decreased by newly developed nanoemulsion quercetin. In the line of decreased serum and hepatic lipid profiles, quercetin-loaded nanoemulsion significantly increased fecal cholesterol excretion in rats fed a high-cholesterol diet. Discrepancy in lipid-lowering effect might result in the incorporation of quercetin into food-grade biopolymer-based nanocarrier used in the current study which leading to the improvement of water solubility and bioavailability of quercetin and protection of degradation [30]. Hepatic mRNA levels involved in bile acid synthesis and cholesterol efflux, such as CYP7A1, LXRα, ABCA1, ABCG1 and ABCG5/8 were significantly upregulated by NQ whereas quercetin did not. In addition to increased mRNA expression, NQ significantly increased hepatic microsomal activity and protein expression of CYP7A1. Liver X receptor alpha (LXRα), a nuclear hormone receptor, acts as a key sensor of dietary cholesterol by increasing the expression of ATP-binding cassette transporter A1 (ABCA1) and ATP-binding cassette sub-family G member 1 (ABCG1), which are involved in cholesterol efflux [54]. LXRα also upregulates ABCG5/8 in the liver and intestine [55], thus reducing cholesterol absorption and upregulating biliary cholesterol secretion [56]. A nuclear hormone receptor acts as a key sensor of dietary cholesterol, LXRα upregulates ABCA1 and ABCG1 expressions involved in cholesterol efflux in states of cholesterol excess [54]. ABCG5/8 expressed in liver and intestine are also upregulated by LXRα in response to dietary cholesterol [55]. Overexpression of human ABCG5 and ABCG8 reduces cholesterol absorption and upregulates biliary cholesterol secretion [56]. Disrupted ABCG5/8 in mice promotes absorption of dietary plant sterols and decreased biliary cholesterol concentrations [57]. Another key enzyme in cholesterol homeostasis, CYP7A1 is the rate-limiting enzyme in bile acid biosynthetic pathways. Upon excessive cholesterol, LXRα upregulates CYP7A1 expression which promotes bile acid synthesis and cholesterol excretion [58,59]. CYP7A1 mutation leads to hypercholesterolemic phenotype with decreased bile acid excretion [60]. Overexpression of CYP7A1 promotes hepatic bile acid synthesis and secretion and increases fecal cholesterol levels [61]. Based on close association between LXRα and cholesterol efflux and between CYP7A1 and bile acid synthesis, our findings suggest that quercetin-loaded double layer nanoemulsion has promising hypocholesterolemic effects compared to quercetin through the activation of LXRα and its-associated ABCG5/8 expression and hepatic microsomal CYP7A1 activity and gene expression. 

In summary, quercetin was newly formulated as a functional delivery system by using oil-in-water nanoemulsion techniques with complexation and self-assembly using caprylic/capric triglyceride (Captex^®^ 355), polyoxyethylene sorbitan monooleate (Tween 80), sodium alginate and soy lecithin. At 6.5, 7.0, 7.5, 8.0, 8.5 and 9.0 pH levels, nanoemulsions showed a good stability showing dispersibility and transparency without any occurrence of flocculation, clump, separation and opaque. Compared to acidic pH levels, smaller monomodal particle size, acceptable range of PDI, less than −30 mV zeta potential values and long term storage stability were observed at pH between 6.5 and 9.0. Therefore, quercetin-loaded oil-in-water nanoemulsions prepared at pH range between 6.5 and 9.0 were stable. Next, the hypocholesterolemic effects of nanoquercetin were investigated in rats fed a high-cholesterol diet. 4-week supplementation with nanoemulsion quercetin ameliorates HC diet-induced serum and hepatic lipid abnormalities and increases fecal lipid excretion, as well as fecal bile acid content in rats fed a high cholesterol diet. In addition, nanoemulsion quercetin significantly upregulates gene expression involved in cholesterol efflux and mRNA level and activity of CYP7A1 related to bile acid synthesis in liver tissue. Thus, our findings suggest that consumption of dietary nanoemulsion quercetin might be a promising application to prevent and/or treat dyslipidemia and its-associated CVD.

## Figures and Tables

**Table 1:** Effect of pH on the appearance and characterization of quercetin-loaded oil-in water nanoemulsion.

pH	Appearance of Nanoemulsion Formation					Characterization of Nanoemulsion Quercetin		
	Dispersed	Flocculated	Clump & Separated	Opaque	Transparent	Particle Size (nm)	Polydispersity Index	Zeta Potential (mV)
**4.0**	+	++	++	+	+	289 ± 3	0.47 ± 0.03	−22 ± 4
**4.5**	+	++	++	+	+	223 ± 2	0.41 ± 0.01	−32 ± 3
**5.0**	+	++	++	+	+	226 ± 3	0.38 ± 0.04	−38 ± 6
**5.5**	+	++	++	+	+	242 ± 6	0.38 ± 0.02	−45 ± 1
**6.0**	+	+	+	+	+	244 ± 7	0.39 ± 0.03	−52 ± 3
**6.5**	++	-	-	-	++	212 ± 1	0.30 ± 0.02	−61 ± 5
**7.0**	++	-	-	-	++	211 ± 1	0.29 ± 0.02	−62 ± 4
**7.5**	++	-	-	-	++	207 ± 2	0.27 ± 0.01	−65 ± 2
**8.0**	++	-	-	-	++	217 ± 2	0.28 ± 0.01	−65 ± 2
**8.5**	++	-	-	-	++	210 ± 1	0.27 ± 0.01	−66 ± 2
**9.0**	++	-	-	-	++	219 ± 4	0.34 ± 0.02	−62 ± 3

Visual appearance of quercetin-loaded nanoemulsions were defined as disperse (+, undispersed; ++, dispersed), flocculation (-, not flocculated; +, partially flocculated, ++ flocculated), clump (-, not clumped; + partially clumped; ++ clumped), separation (-, not separated; + partially separated; ++ separated), opaque (-, not opaque; + opaque) and transparency (+, not transparent; ++ transparent).

**Table 2:** Effects of quercetin and quercetin nanoemulsion on physiological variables.

Group	NOR	HC	LQ	HQ	LNQ	HNQ
Initial body weight (g)	215.4 ± 7.6	215.7 ± 7.9	215.8 ± 8.4	216.0 ± 8.4	216.5 ± 10.8	216.5 ± 9.7
Final body weight (g)	392.7 ± 22.5	389.7 ± 38.9	375.6 ± 33.1	375.6 ± 33.1	373.6 ± 34.7	393.3 ± 26.8
Body weight gain (g/4 week)	177.2 ± 22.0	174.0 ± 32.2	159.8 ± 29.2	159.8 ± 29.2	157.1 ± 31.5	176.8 ± 19.3
Food intake (g/day)	25.2 ± 1.6	23.7 ± 2.3	23.9 ± 2.2	23.9 ± 2.2	23.5 ± 2.3	24.5 ± 1.5
Food efficiency (g gained/kcal consumed)	0.25 ± 0.02	0.26 ± 0.03	0.24 ± 0.03	0.24 ± 0.03	0.24 ± 0.03	0.26 ± 0.02
Epididymal fat weight (g/100g body weight)	1.58 ± 0.30	1.30 ± 0.24	1.45 ± 0.27	1.29 ± 0.39	1.35 ± 0.45	1.43 ± 0.25
Liver weight (g/100g body weight)	2.99 ± 0.09 ^a^	4.43 ± 0.17 ^b^	4.40 ± 0.16 ^b^	4.35 ± 0.09 ^b^	4.67 ± 0.21 ^b^	4.66 ± 0.13 ^b^
Serum GOT (IU/L)	45.75 ± 3.10	50.45 ± 4.18	48.86 ± 4.76	42.70 ± 2.19	50.98 ± 2.71	50.77 ± 3.49
Serum GPT (IU/L)	7.62 ± 0.83	7.29 ± 0.61	7.42 ± 1.08	6.07 ± 0.77	7.58 ± 0.73	7.01 ± 0.78

Values are expressed as mean ± SEM (*n* = 8). ^a,b^ Mean values with unlike superscript letters are significantly different at *p* < 0.001 level by Tukey’s multiple range test. NOR: normal chow diet, HC: high cholesterol diet containing 1% cholesterol and 0.5% cholic acid, LQ: HC containing 0.05% quercetin, HQ: HC containing 0.1% quercetin, LNQ: HC containing 0.05% quercetin nanoemulsion, HNQ: HC containing 0.1% quercetin nanoemulsion.

## Supplementary Materials

The following are available online at http://www.mdpi.com/2072-6643/11/2/244/s1, Figure S1: Schematic illustration of possible incorporation of quercetin in oil-in-water nanoemulsion, Figure S2: Visual appearance of quercetin-loaded nanoemulsions at (**A**) 21 °C and (**B**) 37 °C for 3-month storage, Figure S3: Correlation between zeta potential and encapsulation efficiency for quercetin-loaded nanoemulsions, Figure S4: Transmission electron microscope image of quercetin nanoemulsion used in animal study (scale bar = 50 nm), Table S1: Compositions of experimental diets (units: g/kg diet), Table S2: Primers used for real-time qPCR.
