# Supplementary material for: Formulation and Characterization of Quercetin-loaded Oil in Water Nanoemulsion and Evaluation of Hypocholesterolemic Activity in Rats
Source: Nutrients. 2019 Jan 22;11(2):244. doi: 10.3390/nu11020244 (PMC6412563; doi:10.3390/nu11020244)
Supplement: Supplementary file 1 [file nutrients-11-00244-s001.pdf]

nutrients

Table S1. Compositions of experimental diets (units: g/kg diet).

Component	HC	LQ	HQ	LNQ	HNQ
Corn starch	150.0	150.0	150.0	150.0	150.0
Casein	200.0	200.0	200.0	200.0	200.0
Sucrose	485.0	484.5	484.0	484.5	484.0
Corn oil	50.0	50.0	50.0	50.0	50.0
Cellulose	50.0	50.0	50.0	50.0	50.0
Mineral mix [a]	35.0	35.0	35.0	35.0	35.0
Vitamin mix [b]	10.0	10.0	10.0	10.0	10.0
DL-Methionine	3.0	3.0	3.0	3.0	3.0
Choline bitartrate	2.0	2.0	2.0	2.0	2.0
Experimental Powder	0.0	0.5	1.0	0.5	1.0
Cholesterol	10.0	10.0	10.0	10.0	10.0
Cholic acid	5.0	5.0	5.0	5.0	5.0
Total	1,000	1,000	1,000	1,000	1,000
Total Calorie (kcal)	3,714	3,712	3,710	3,712	3,710
Carbohydrate (% as kcal)	66.8	66.8	66.7	66.8	66.7
Protein (% as kcal)	19.6	19.6	19.6	19.6	19.6
Fat (% as kcal)	12.1	12.1	12.1	12.1	12.1

HC: high cholesterol diet containing 1% cholesterol and 0.5% cholic acid, LQ: HC containing 0.05% quercetin, HQ: HC containing 0.1% quercetin, LNQ: HC containing 0.05% quercetin nanomeulsion, HNQ: HC containing 0.1% quercetin nanoemulsion.
Experimental diets were transformed based on the AIN -76 diet composition.
[a] AIN-76 Mineral mix.
[B] AIN-76 Vitamin mix.

Table S2. Primers used for real-time qPCR.

Name	Genbank No.	Primer sequence (5′ - 3′)	Amplicon size (bp)
ABCG5	NM_053754	F: ATGAGTGAGCTGCCCTTTCT<br>R: CGCTGAAGGACACATTCAGG	142
ABCG8	NM_130414	F: GAGAACTTTGTCCCGCCTGG<br>R: AGATAGGGGTGCCAGACGTC	122
β-actin	NM_031144	F: GGCACCACACTTTCTACAAT<br>R: AGGTCTCAAACATGATCTGG	123
CYP7A1	NM_012942	F: TGTTCTGTGTTCACTTTCTG<br>R: ACTCGGTAACAGAAGGCATA	126
LXRα	NM_031627	F: GACTTCGAGTCACGCCTTGG<br>R: GTCCTCCCTGCTCAGCTGTA	161

ABCG5, ATP-binding cassette sub-family G member 5; ABCG8, ATP-binding cassette sub-family G member 8; CYP7A1, Cholesterol 7 alpha-hydroxylase; LXRα, liver X receptor α.

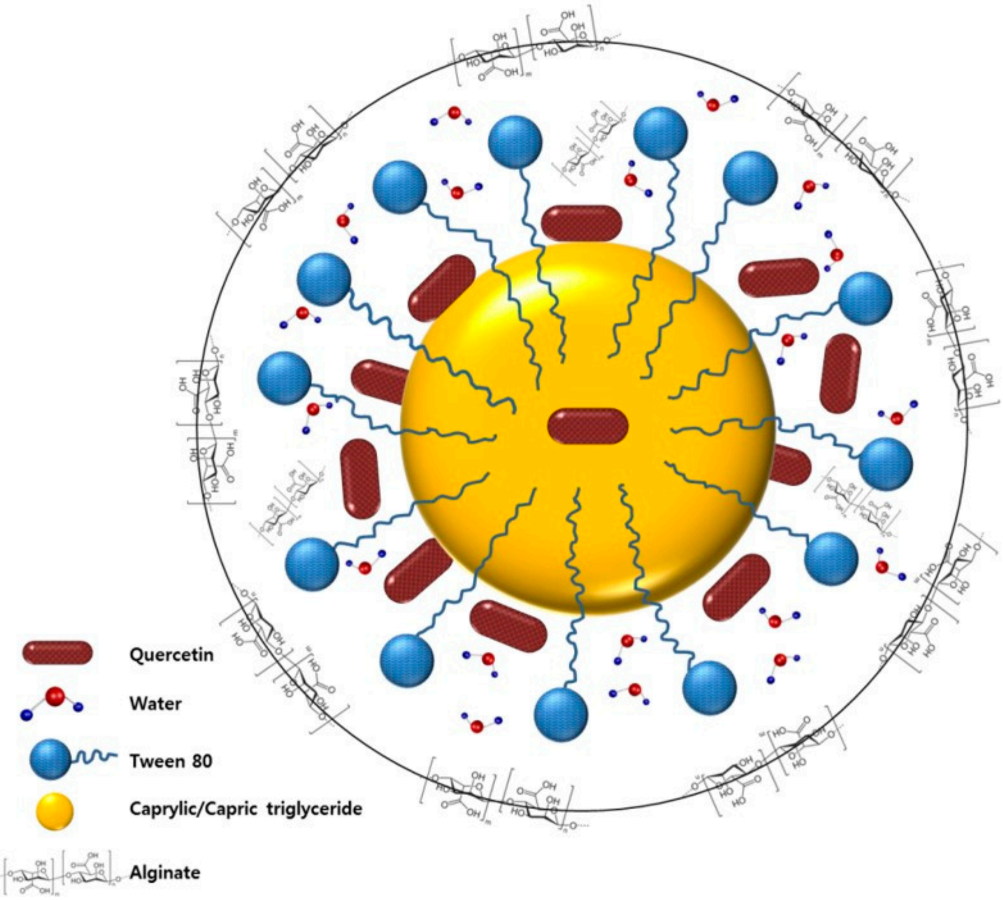

Figure S1. Schematic illustration of possible incorporation of quercetin in oil-in-water nanoemulsion.

A

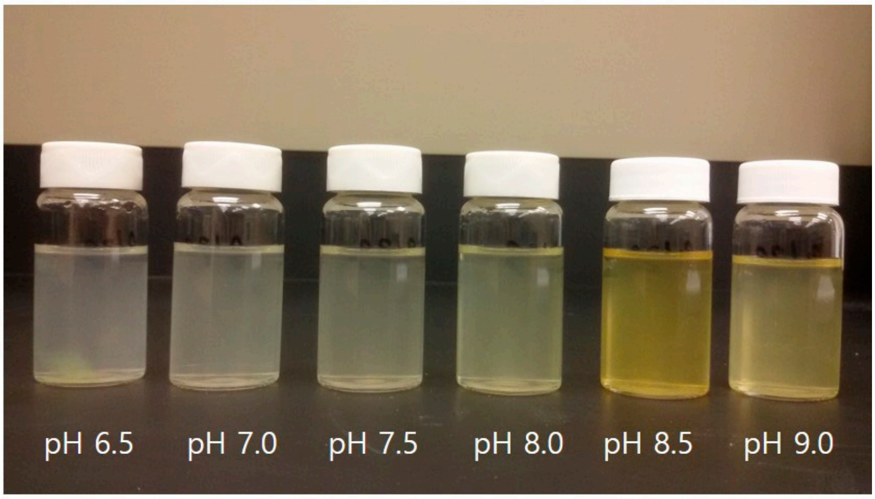

B

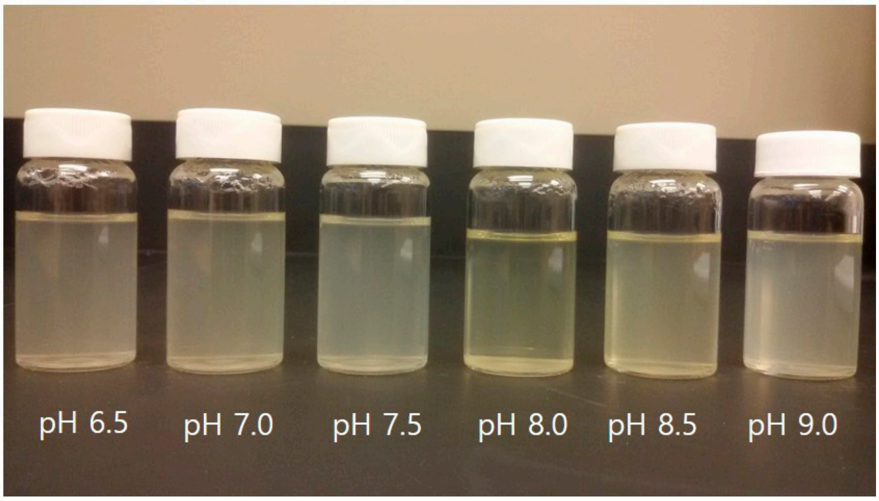

Figure S2. Visual appearance of quercetin-loaded nanoemulsions at (**A**) 21 °C and (**B**) 37 °C for 3 month storage.

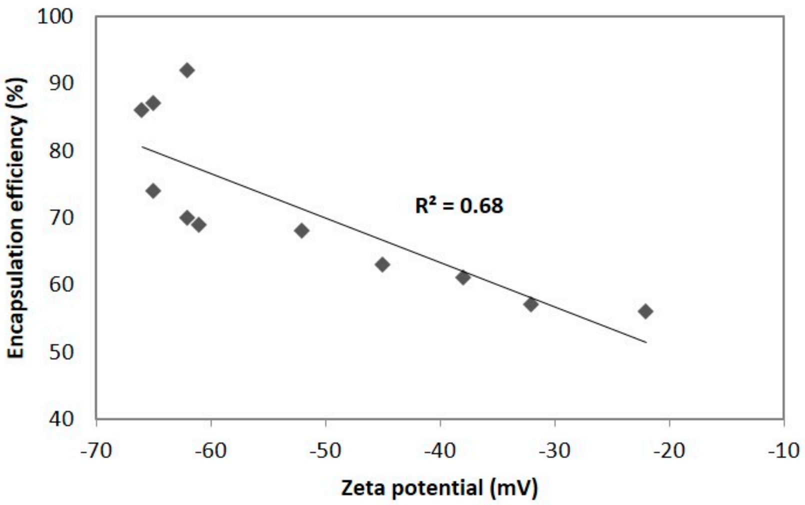

Figure S3. Correlation between zeta potential and encapsulation efficiency for quercetin-loaded nanoemulsions.

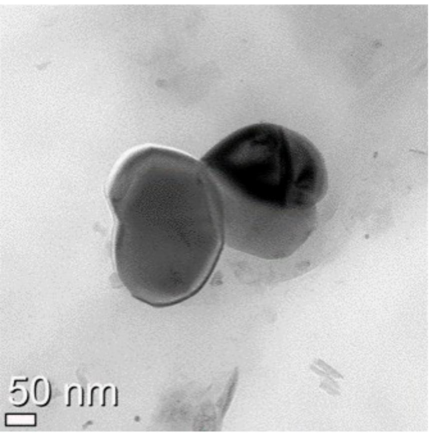

Figure S4. Transmission electron microscope image of quercetin nanomeulsion used in animal study (scale bar = 50 nm).

© 2018 by the authors. Submitted for possible open access publication under the terms and conditions of the Creative Commons Attribution (CC BY) license (http://creativecommons.org/licenses/by/4.0/).
